# A case for monitoring fungi of clinical relevance at beaches: enterococci measures do not correlate with all disease risks

**DOI:** 10.1007/s10661-025-14909-3

**Published:** 2026-01-13

**Authors:** Larissa Montás-Bravo, Helena M. Solo-Gabriele, Débora F. Gil, Ayaaz Amirali, Sebastian P. Arenas, Sofia Hoffman, Adriana Jimenez, Alexandre Mendonça, Rivka Reiner, Raquel Sabino, Konstantina Sarioglou, Mark E. Sharkey, Bhavarth S. Shukla, Isabella J. Tavarez, Elisabete Valério, Cristina Veríssimo, João Brandão

**Affiliations:** 1 Department of Chemical, Environmental, and Materials Engineering, University of Miami, Coral Gables, FL 33146 USA; 2 Department of Environmental Health, National Institute of Health Doctor Ricardo Jorge, Lisbon, Portugal; 3 University of Miami Health System, Miami, FL USA; 4 Department of Public Health Sciences, University of Miami Miller School of Medicine, Miami, FL USA; 5 CBMA (Center of Molecular and Environmental Biology), Department of Biology, University of Minho, Braga, Portugal; 6 Research Institute for Medicines (iMed.ULisboa), Faculty of Pharmacy, Universidade de Lisboa, Lisbon, Portugal; 7 Environmental Health Institute, Faculty of Medicine, Universidade de Lisboa, Lisbon, Portugal; 8 Laboratório Associado TERRA, Instituto Superior de Agronomia, Universidade de Lisboa, Lisbon, Portugal; 9 Department of Medicine, University of Miami Miller School of Medicine, Miami, FL USA; 10 CE3C Centre for Ecology, Evolution and Environmental Changes, Faculdade de Ciências da Universidade de Lisboa, Lisbon, Portugal; 11 Department of Infectious Diseases, National Institute of Health Doctor Ricardo Jorge, Lisbon, Portugal; 12 Research Data and Open Scholarship, UM Libraries, University of Miami, Coral Gables, FL 33146 USA

**Keywords:** Fungi, Beaches, *Aspergillus*, *Candida*, Sand, Pathogens

## Abstract

Fungal disease is on the rise, coupled with fungal pathogens increasing in geographic range. Studies have shown that viable fungal pathogens may be present in beach sand and water, and consequently, efforts are ongoing in Europe to develop guidelines for fungi levels at beaches. In the USA, fungal diseases are a growing concern, and yet, they are not subject to public health reporting, and beach environments are currently not routinely monitored for fungal pathogens. This study measured fungal and enterococci levels at two beaches within a subtropical environment in Miami, FL. Samples were analyzed by culture-based methods, with fungi species confirmation by targeted PCR and sequencing. A unique aspect of this study is the analysis with higher incubation temperatures (37 °C and 40 °C) to isolate mesophilic fungi that can cause invasive infections. Results show that levels of fungi were strongly influenced by sample matrix (sand or water). The most common fungal species observed belonged to the *Aspergillus* and *Candida* genera, with the isolation of 25 taxa with reports of causing infections, of which 20 were previously reported to exhibit resistance to some or all classes of antifungals available. Results emphasize the need for fungal specific analyses to better understand beach-related disease risks. Given the current increase in cases of fungal diseases and the presence of viable fungi in the environment, integrating fungal measurements in routine microbiological monitoring programs is critical for assessing the beach transmission of pathogenic fungal disease and the control of emerging fungal pathogens.

## Introduction

Many experts acknowledge that fungal disease is on the rise, coupled with fungal pathogens increasing in geographic range. The World Health Organization estimates that more than 300 million people worldwide may be affected by serious fungal pathogens each year, potentially causing more than 1.5 million deaths annually, and in the USA, studies have found fungal disease is a serious clinical concern, with substantial healthcare costs and significant increases in morbidity and mortality (WHO 2022; Casadevall et al., 2019; Rayens & Norris, 2022). However, most fungal diseases are not subject to public health reporting requirements, and fungal pathogens are not included in environmental surveillance of potential human exposure areas (CDC 2024). Because of the limited public health and environmental surveillance, it is not clear how people are getting exposed to fungal pathogens and what factors determine disease prevalence.

Fungal pathogens can cause infections in various parts of the body. Fungal pathogens can enter the human body through cuts, wounds, burns, and inhalation (Shah et al., 2024). Fungal illnesses vary in the level of tissue involvement, ranging from superficial to invasive infections. Dermatophytes, such as *Microsporum*, *Epidermophyton*, and *Arthroderma*, mainly infect hair, skin, and nails (Moskaluk & VandeWoude 2022). Other fungal groups may cause highly invasive, deep, and/or subcutaneous infections. Disseminated infection can be life-threatening and can result in hematological and pulmonary infections. Pathogenic fungi may be shed by skin, feces, or respiratory secretions. Additionally, pathogenic fungi may be found on different surfaces, in soil, and in other media, resulting in transmission via direct contact or inhalation (Reddy et al., 2022).

One area for potential exposure to fungal pathogens is the beach environment. Larrondo and Calvo (1989) determined that Mediterranean coast beach sands are a suitable media that maintains viable keratinophilic fungi. A study by Salvo and Fabiano (2007) found opportunistic fungal pathogens and pathogenic filamentous fungi in Lugurian beach sediments during the tourist season. Thus, beach sand can be a reservoir of fungal species with pathogenic capacity. Beach sand is a habitat that supports many microbes, including bacteria, viruses, fungi, and protozoa. These microbes are found in the sand’s interstitial spaces or adhered to the sand grains (Whitman et al., 2014). Marine water also supports various bacteria, viruses, and fungi. There is a complex connection between the beach sand and water microbiome which plays a crucial role in the microbial distribution in each matrix. Marine water may be polluted by stormwater runoff, sewage spills, riverine discharge, and coastal circulation (Tilburg et al., 2015; Montas Bravo et al., 2024). Humans and animals that frequent the beach can inoculate the sand through contact (Abreu et al., 2016; Deligios et al., 2023). In addition, the sand can be inoculated from the water through tidal flows, waves (Feng et al., 2016), and aerosols generated from breaking waves (Abdool-Ghany et al., 2023). Once the beach sand is contaminated by fungi, these microbes can multiply to colonize it. Fungi adapt to different environmental conditions at the cellular and molecular level, allowing them to survive and grow in high ambient temperature and high salinity. Several studies have investigated the underlying mechanisms, at the cellular and molecular level, of fungi adapting to changing environmental conditions (Plemenitas et al., 2014; Wani et al., 2022; Gostinčar et al., 2024). Thus, many fungi species are mesophile and halotolerant and organisms: they remain viable and replicate in high temperature and high salinity environments (Gunde-Cimerman et al., 2018; Boumaaza et al., 2022). The humidity and nutrients retained in beach sand represent an optimal environment for fungi proliferation. Beaches are also places where a larger surface area of the human body is in intimate contact with sand and water; thus, there is a possibility of greater exposure to fungi (Romão et al., 2015).

Potential impacts to human health from pathogenic microbes in the environment are derived by monitoring human exposure zones, such as the beach environment. The results from analysis of different microbial pathogens inform on risks and their potential impact on human health. The recent spread of emerging fungal pathogens in the USA prompts the need to assess whether current environmental monitoring practices (focused on measuring fecal indicator bacteria (FIB) levels in recreational water and are not inclusive of fungi) capture all microbial threats to successfully inform public health advisories. In the USA, the FIB group enterococci are monitored at beaches to assess water quality and potential health risks from recreational exposure, but are not monitored in sand. Enterococci have been adopted as indicators of human fecal pollution in water due to their ubiquity in human feces and persistence in water. Studies have found that enterococci can be found in shoreline sediments and can persist within catch basins and wells in stormwater conveyance systems due to runoff contaminated by urban sediments, human and animal waste (Montas Bravo et al., 2024). Recent studies have questioned the validity of using enterococci as the single indicator of the presence of pathogens causative of various human illness, as they may not always correlate with actual pathogen presence (Hayes et al., 2023; McKee & Cruz, 2021), leading to studies advocating the use of alternative indicators for water quality assessments (McLellan & Eren 2014). Currently, in the USA, enterococci are not monitored in sand.

The objective of this study was to measure and compare enterococci and fungal levels (water and beach sand) at two study beaches within a subtropical environment (Miami, FL) and to recommend an approach for integrating fungal measurements into routine monitoring programs based upon these measurements. Our study is novel because it compares fungal results with FIB, which has not been done before. The only study that evaluated FIB and fungi evaluated only total fungi and did not document the fungal species (Carducci et al., 2022). Moreover, a unique aspect of this study is the use of higher incubation temperatures to isolate fungi that can cause invasive infections. In the USA, the emphasis has been on monitoring FIB. Studies that evaluate fungi in the USA are rare and have not made comparisons against the FIB measures to emphasize that FIB cannot provide an indication of fungi contamination and thus do not inform on all potential microbial risks to human health. Both enterococci and fungi species are halotolerant and mesophile organisms: they persist and grow in high salinity environments with moderate temperatures (20 to 45 °C). The inclusion of enterococci measurements allowed for a comparison between fungi and FIB as a first step in evaluating whether enterococci-based measurements correlate with fungi levels in beach environments. The results of this study can be used to justify the limitations of relying exclusively on enterococci measurements to protect human health at beaches.

## Methods

### Study site and sample collection

**Fig. 1:**
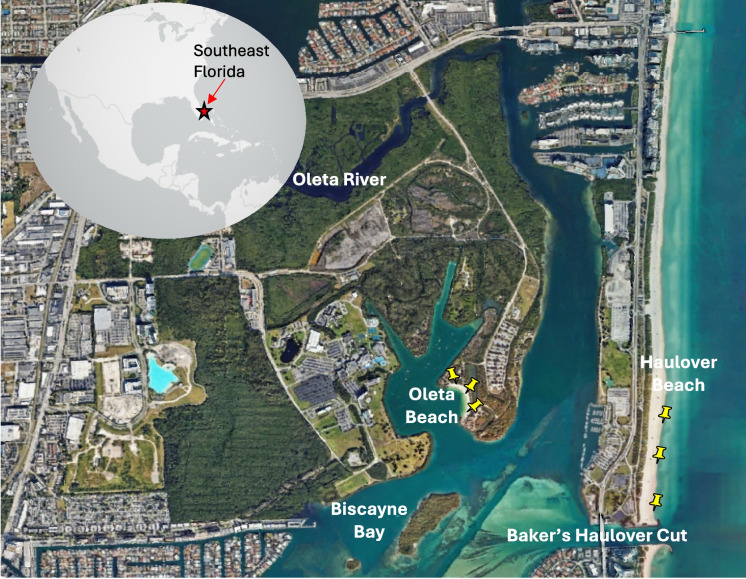
Location of beaches (Haulover and Oleta) that were evaluated in this study. Yellow pins show locations of south, middle, and north sampling sites at each beach

The two marine beach sites chosen for this study are located in Miami-Dade County, Florida, USA. Haulover Beach (25° 54′ 5.4″N, 80° 7′ 18″W) is an ocean-facing beach located immediately north of Baker’s Haulover Cut, a man-made inlet that connects northern Biscayne Bay with the Atlantic Ocean. A stone pier delimits the southernmost part of Haulover Beach, which extends approximately 1 km (Fig. 1). Oleta Beach (25° 54′ 29.2″N, 80° 7′ 57.4″W) faces a small lagoon nested within the Oleta River State Park, a natural preserve surrounded by mangroves.

Sample collection for this study occurred during the summer months at high tide on three separate dates, on June 2, August 2, and August 31, 2023. The sites are in an area characterized by wet versus dry seasons. The summer months correspond to the wet season with a total cumulative rainfall during the three sampling months (June 1 to August 31) of 64.9 cm (NOAA Station USW00012839). During sample collection, ambient conditions were recorded using visual observations (for the number of people and dogs at the beach) and from a mobile iPhone app (NavClock, 4.4.2, Split Rail, Inc.) which interpolates air temperature and wind conditions from local weather stations. Basic water quality was measured using a pre-calibrated water quality sonde (YSI DSS Pro, Yellow Springs, OH) capable of measuring water temperature, pH, salinity, turbidity, and dissolved oxygen. The surface temperature of the sand was measured using a handheld laser thermometer (MT, Raytek®).

All sampling occurred during the early morning hours (8 am to 10 am). Sand was collected from the supratidal zone at three equidistant locations across the beach site (Fig. 1), following the protocol of the Mycosands II initiative, which aimed to provide data that captures the variation along the length of a designated beach. Samples were collected from each location from the top 10 cm using a sterile spoon and placed into separate sterile Whirlpak™ bags. A surface water sample was collected from the center of the beach in waist-deep water (1 m depth). The sterile bottle was rinsed three times, and the bottle was immersed a few centimeters below the surface at a location upstream of the sampler. Upon collection, all samples were placed in a cooler containing ice packs. Samples were processed at the laboratory within 2 h of collection.

### Laboratory methods

To provide a basis for comparison with current US recommended approaches for assessing the microbial quality of recreational marine beaches, this study evaluated both enterococci and fungi. The fungal analysis methods are those of the Mycosands I (Brandão et al., 2021) and II; international studies focused on establishing baseline levels of fungi in beach environments with an emphasis in Europe.

### Enterococci in water and sediments

Upon receipt at the laboratory, water samples were analyzed for enterococci using a chromogenic substrate approach. In brief, the method involved diluting a 10-mL sample with 90 mL of phosphate buffered saline (PBS) and then adding Enterolert™ reagent (IDEXX Industries) until dissolution. The mixture was then added to trays, Quantitray-2000,that allowed for enumeration based upon most probable number (MPN). Wells that fluoresced under UV light, after 24 h of incubation at 41.5 °C, were identified as positive. Sediments were processed similarly as for water. Sediment required preprocessing by mixing the sand to homogenize samples from the three sampling locations at the corresponding beach site. An aliquot of the composite sand sample was removed for moisture content by gravimetric analysis (dried at 100 °C for 24 h) so that the results could be normalized on a per dry weight basis. Another aliquot (about 10 g) was eluted with 100 mL of PBS by shaking as per the protocol recommended by Boehm et al. (2009). Ten milliliters of this elution was processed like the water samples by chromogenic substrate as described above.

### Fungi in water and sediments

A unique aspect of this study is the focus on risks to human health from pathogenic fungi. Thus, we used Sabouraud Agar (Salt Sabouraud Dulcitol Agar, with CAM and GEN, 90 mm, Thomas Scientific) (SDA) plates and Mycosel Agar (Thermo Scientific™ Mycobiotic Agar with Chloramphenicol and Cycloheximide, 90 mm) (MCA) plates. Sabouraud is known to work well for the recovery of fungi from the environment (Sandven & Lassen, 1999), and Mycosel agar is the standard medium for the isolation of dermatophytes (Shadomy & Philpot, 1980), since the presence of cycloheximide inhibits the saprophytic fungi with a high growth rate.

**Fig. 2:**
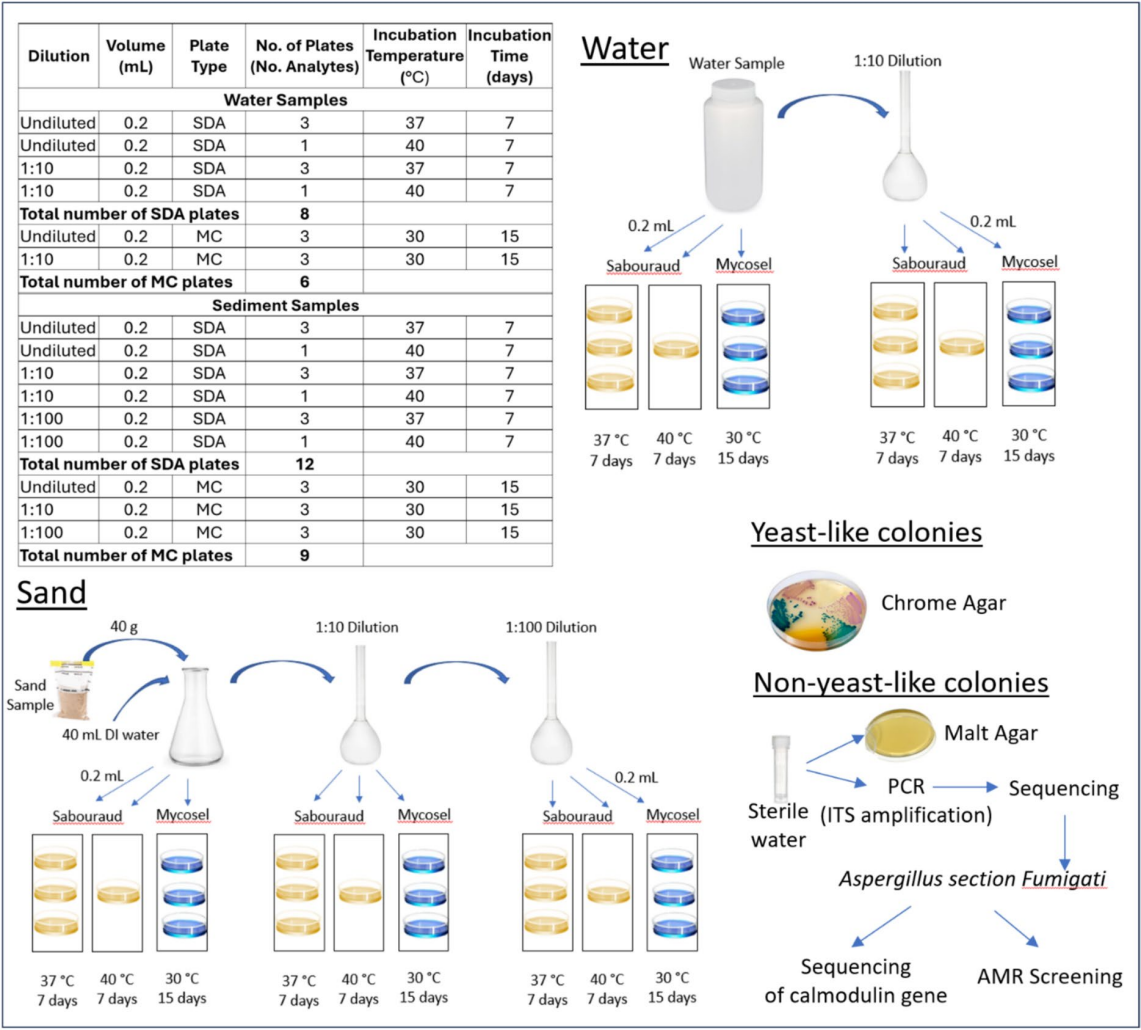
Laboratory processing for water and sediment samples. Incubation temperature of 37 °C used to select for human pathogens and incubation temperature of 40 °C used to select for highly thermotolerant *Candida* spp.

Analysis was conducted by pipetting 0.2-mL undiluted, and 0.2 mL of a 10:1 dilution, water samples directly and spreading onto a total of eight Sabouraud plates and six Mycosel plates per water sample for each of the three sampling days (Fig. 2). The Sabouraud plates (in triplicate) were incubated at 37 °C for 7 days. Similarly, the Mycosel plates (in triplicate) were incubated for 15 days at 30 °C. The additional two Sabouraud plates were used to process 0.2 mL of an undiluted and a 1:10 dilution of the sample which were incubated at 40 °C for 7 days. This higher incubation temperature was used to select highly thermotolerant *Candida* spp., in particular *C. auris*. The higher incubation temperatures of Sabouraud plates at 37 °C and 40 °C (usually 27.5 °C) are unique to this study and important, as this higher temperature isolates mesophilic fungi that can cause invasive infections which are more relevant to human health.

Fungal analysis in sediments followed a similar process as water. However, the samples were first pre-processed to elute the fungi from the sediment. Elution was performed by processing a 40-g sample of composite sand and adding 40 mL of sterile deionized water and swirling the water gently to minimize the breakage of fungal hyphae, which would artificially increase the number of colony-forming units, resulting in higher fungal counts. Like water, 0.2 mL of undiluted wash water and a 1:10 dilution of wash water (diluted in PBS) were added to eight Sabouraud plates and to six Mycosel plates as described above. However, due to the heavy growth of fungi observed on the plates during the first day of sampling, a third dilution of 1:100 was added for samples processed from the second and third day of sampling (12 Sabouraud plates and 9 Mycosel plates).

Following the different incubation periods described above, all morphologically unique isolates were identified through two methods (Fig. 2, bottom right panel). First, colonies that appeared as yeast (shiny and circular white- or cream-colored colonies) were streaked onto HardyCHROM™ *Candida* + *auris* plates as a medium for primary, selective isolation and differential identification of *Candida* species. Species of *Candida* that grew on the plates were identified based upon color and fluorescence as per manufacturer instructions. Non-yeast-like colonies were inoculated into a sterile 2-mL centrifuge tube filled with sterile deionized water. These isolates were shipped from the University of Miami to the Department of Environmental Health of the National Institute of Health Doutor Ricardo Jorge (UTI-INSA) in Lisbon, Portugal, for identification by targeted sequencing and for further analysis of *Aspergillus section Fumigati* complex.

At the UTI-INSA, most isolates were identified according to their morphological features after growth on Malt plates. However, some isolates posed some doubts about their identification and were therefore identified using molecular biology methods, through sequencing of the ITS region. For the selected isolates, a small portion of the colony was scraped, and the total DNA was extracted using Dneasy UltraClean kit (Qiagen) according to manufacturer’s instructions. In a first step, the entire ITS region and part of the adjacent 18S and 28S genes were amplified using primers ITS5 (5′-GGAAGTAAAAGTCGTAACAAGG) and LR6 (5′-CGCCAGTTCTGCTTACC) yielding an amplicon of ca. 900 bp (Hoang et al., 2019). The reactions were performed in a thermocycler (Biometra) with a hot lid (95 °C) using the following conditions: initial denaturation at 95 °C for 5 min, followed by 40 cycles of 95 °C for 30 s, 60 °C for 11 min, and 72 °C for 1 min and 30 s, with a final extension at 72 °C for 5 min (Valerio et al., 2008). To confirm the DNA amplification, the PCR products were resolved by electrophoresis in 1% (w/v) agarose gels at 75 V for 45 min in 1 × TBE buffer, where GelRed was incorporated in the gel to allow the visualization of the PCR amplicons. The gel image was acquired using a gel transilluminator (UVITEC) under UV light. The amplified products were purified using the ExoProStar kit to remove residual primers and unincorporated nucleotides, guaranteeing clean DNA for downstream applications (5 μL of PCR product was added to 2 μL of ExoProStar 1-Step). The mixture was then placed in a thermocycler for 15 min at 37 °C to allow for enzymatic digestion of unwanted components, followed by a 15-min incubation at 80 °C to inactivate the enzymes, thus ensuring that the purified DNA was ready for further analysis (Cytiva, 2013).

The second step used purified PCR products to obtain the ITS region sequence. In brief, two separate amplification reactions were prepared for each sample, one with the ITS1 forward primer ITS1 (5′-TCCGTAGGTGAACCTGCGG) and the other with the ITS4 reverse primer ITS4 (5′-TCCTCCGCTTATTGATATGC). Each reaction had a total volume of 10 μL consisting of 1 μL of the purified PCR product, 1 μL of the sequencing primer (ITS1 or ITS4), 1 μL of BigDye buffer, 1 μL of BigDye Terminator v3.1 Cycle Sequencing Mix (Applied Biosystems), and 6 μL of sterile water. The sequencing PCR was carried out with the following cycling conditions: an initial denaturation at 96 °C for 10 s, followed by 25 cycles of 50 °C for 5 s, and 60 °C for 4 min. The final extension step was conducted at 72 °C for 10 min. These conditions ensured accurate amplification and extension of the DNA fragments for subsequent sequencing analysis (Valerio et al., 2008).

The samples were then sent for sequencing at UTI-INSA. Sequencing was conducted in both directions using BigDye Terminator v1.1 Cycle Sequencing Kit (Applied Biosystems). Dye terminators were removed from sequencing reactions with DyeEx 96 plates (QIAGEN) according to the manufacturer’s instructions; the eluted products were dried at 70 °C in a thermocycler and subsequently resuspended in 15 μL HiDi formamide (Applied Biosystems). Electrophoretic separation of sequencing products was performed using POP-7 polymer on a 3130xl Genetic Analyzer equipped with a 50-cm capillary array (Applied Biosystems).

The chromatograms from the sequencer were read and edited using the BioEdit program (Hall 1999). The sequences were analyzed with the GenBank nucleotide data library using a BLASTn search (https://blast.ncbi.nlm.nih.gov/Blast.cgi) to confirm the reliability of the ITS sequences and determine their closest relatives. Species identification was accepted when the homology was ≥ 98%. If the similarity was between > 60% and < 97%, only the genus was considered for identification. The ITS region sequences obtained were deposited in GenBank (accession numbers PQ834260, PQ834261, PQ834262, PQ834263, and PQ834264).

For further analysis of *Aspergillus fumigatus* complex for possible cryptic species identification, partial sequencing of the gene encoding calmodulin was performed using the primers cmd5 (5′-CCGAGTACAAGGAGGCCTTC-3′) and cmd6 (5′-CCGATAGAGGTCATAACGTGG-3′) as described previously by Sabino et al. (2014a, b) and as follows: amplifications were performed in a 25 μL volume reaction of PCR beads (Illustra PuReTaq Read-to-Go; GE Healthcare, Buckinghamshire, UK), containing 15 pmol of each primer and 20–50 ng of Aspergillus genomic DNA. Amplifications were carried out with an initial denaturation at 95 °C for 10 min, followed by 38 cycles of 95 °C for 30 s, 55 °C for 30 s, and 72 °C for 1 min, followed by a final extension step of 72 °C for 7 min. PCR products were analyzed by electrophoresis through 2% agarose gels. The resultant PCR amplicons were purified using the ExoSAP-IT enzyme system (USB Corporation, Cleveland, OH, USA), according to the manufacturer’s instructions (incubation at 37 °C for 15 min, followed by another step of 15 min at 80 °C). Sequencing reactions were performed with the BigDye terminator v 1.1 cycle sequencing kit (Applied Biosystems) in the thermal cycler using the same primers as were used in the PCR amplification and according to the following conditions: an initial denaturation at 96 °C for 5 min, followed by 30 cycles of 96 °C for 10 s, 50 °C for 5 s, and 60 °C for 4 min, followed by a final extension step of 72 °C for 5 min. The resultant nucleotide sequences were edited with Chromas Lite v 2.01 and aligned with CLUSTALX v 2.1 programs. Edited sequences were further compared with sequences deposited in the GenBank (Bethesda, MD, USA) and to achieve the identification to species level, accepted when the obtained homology was ≥ 98%. Sequences were deposited in GeneBank database under the following Gene Bank Accession Numbers: PQ863779—PQ863788.

The resultant nucleotide sequences were edited with Chromas Lite v 2.01 and aligned with CLUSTALX v 2.1 programs. Edited sequences were further compared with sequences deposited in GenBank (Bethesda, MD, USA) and to achieve the identification to species level, accepted when the obtained homology was ≥ 98%. For detecting AMR, specifically azole resistance in *A.* section *Fumigati*, strains (a conidial suspension of 0.5 McFarland) were screened using SDA media supplemented with itraconazole (4 mg/L), voriconazole (2 mg/L), and posaconazole (0.5 g/L), following the clinical breakpoints proposed by the European Committee on Antimicrobial Susceptibility Testing (Meletiadis et al., 2022).

### Data analysis

To assess if fungal levels were associated with enterococci levels, concentrations of microbes observed at the beach sites were compared to regulatory guideline levels, where available. In Florida, to safeguard public health, the regulatory guideline level of enterococci for marine recreational waters is 70 MPN per 100 mL of water sampled (FDEP 2021). The World Health Organization (WHO) indicates that a value of (intestinal enterococci) 40 cfu per 100 mL of water sampled would be associated with low risk of gastrointestinal illness (WHO 2021). Currently, Florida and the USA do not have a guideline level for the safety of beach sand. The recommendation for beach sand from the World Health Organization (WHO) is 60 CFU/g for enterococci (WHO 2021). The WHO also has listed an average for total fungal counts in sands (90 CFU/g), which is used for comparative purposes. The WHO does not provide a guideline level for fungi in water.

Data were analyzed statistically using Excel and SPSS (version 29). Normality of the data was evaluated by the Shapiro–Wilk test, which indicated that the data were normally distributed for the majority of the data sets. Statistical differences between the Haulover and Oleta data sets were therefore evaluated by using *t*-tests at 95% confidence levels. Differences were considered significant for *p* values less than 0.05. Results from triplicate plates and multiple dilutions (within an agar/temperature combination) were averaged across the corresponding sample. The highest concentrations of repeat fungal species identified on different agar/temperature combinations were reported for health protection.

## Results

### Ambient conditions

Average air and water temperatures during sampling were 28.5 °C and 29.5 °C, respectively. Air humidity was 79% on average. Sand temperatures varied with depth. At Haulover, sand temperature varied from 33 °C at the upper 1 cm surface to 28 °C at a 10 cm depth. At Oleta, sand temperature varied from 27 °C at the upper 1 cm to 26 °C at 10 cm depth. On all sampling days, beachgoers were observed within 100 m of the sampling location (16 on average for Haulover and 24 on average for Oleta). Dogs were observed at Haulover (10 on average) but no seagulls. No dogs were observed at Oleta, but seagulls were frequently observed along the shoreline and wading in the water (18 on average). Water at both beaches was characterized by low turbidity (< 0.1 NTU) due to low wave conditions (< 10 cm in height). The average dissolved oxygen levels in the water were 6 mg/L and 7.3 mg/L for Haulover and Oleta, respectively. Water pH for both sites was 8.2. The water salinity at Haulover (34.6 psu) was higher than the salinity at Oleta (21.6 psu).

### Enterococci

**Fig. 3:**
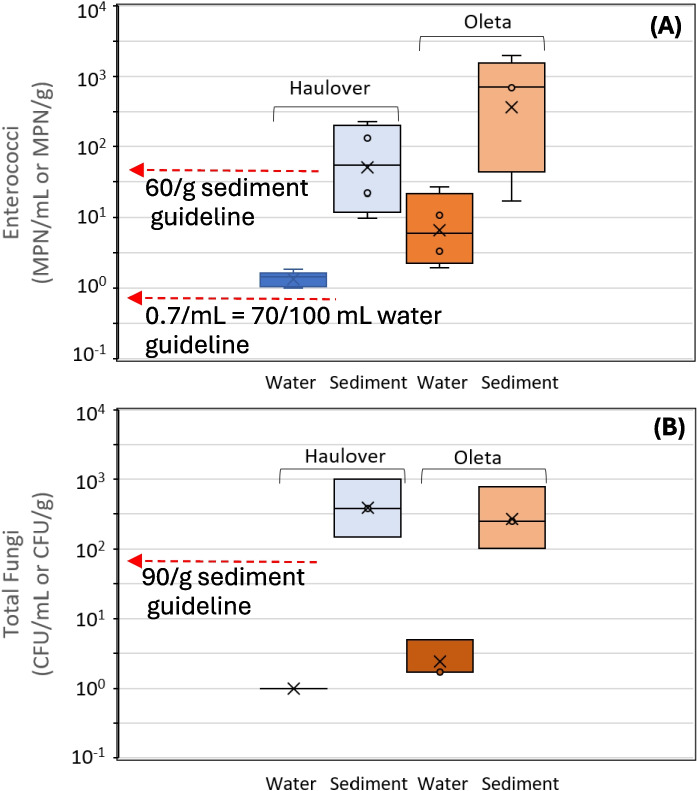
Enterococci (**A**) and total fungal counts (**B**) across beaches and across water and sediments. Note that the traditional units of enterococci count in water were divided by 100 to convert the values from a per 100 mL to a per 1 mL basis

The mean concentration of enterococci, across the three sampling days, in water and sand at Haulover was lower than those at Oleta (Fig. 3 A). Although the enterococci levels were lower at Haulover on average, the levels were not statistically different than those from Oleta (*p* = 0.21 for water, *p* = 0.17 for sand). All water samples collected at both beaches exceeded the local Florida recreational water quality standard of 70 MPN/100 mL for enterococci, (149 MPN/100 mL for Haulover on average, and 1080/100 mL for Oleta on average). Average enterococci levels in sand at Haulover were 98 MPN/g (range from 10 to 130 MPN/g) and at Oleta were 852 MPN/g on average (range from 17 to over 1970 MPN/g). These averages exceeded the 60 MPN/g sand enterococci guideline recommended by the WHO.

### Fungi

Average total fungal counts for sand at Haulover and Oleta Beaches were of the same order of magnitude (1324 CFU/g for Haulover and 1288 CFU/g for Oleta) (Fig. 3B, Table 1) and were not statistically different by beach (*p* = 0.71). The levels observed in beach sand are contrasted with the lower levels found in water (normalizing by mass to compare a per gram to a per mL basis), which had average total counts of 28.4 CFU/mL for Haulover and 20.2 CFU/mL for Oleta Beach. Average total counts at both beaches were a factor of three to five times higher than the average established by the WHO of 90 CFU/g. This study targeted taxa with capacity to cause systemic or invasive infections by utilizing an incubation temperature that potentially reduced fungal counts (by growth inhibition of environmental species at these higher temperatures). However, the WHO guideline averages were based on non-selective incubation temperatures.

**Table 1:** Distribution and levels of fungal species between beaches and between sand and water

	Haulover				Oleta				Pathogenic	Antifungal resistant
	Sand (CFU/g)	DD^b^	Water (CFU/mL)	DD	Sand (CFU/g)	DD	Water (CFU/mL)	DD		
Total	**1324**^a^	**3**	**28.4**	**1**	**1288**	**3**	**20.2**	**3**		
*Alternaria alternata*	-	0	**1.7**	**1**	-	0	**1.7**	**1**	Y	Rarely
*Aspergillus candidus*	**35.0**	**1**	-^c^	0	-	0	-	0	Rarely	N
*Aspergillus* section *Flavi*	**162.5**	**2**	**16.7**	**1**	**26.9**	**3**	-	0	Y	Y
*Aspergillus* section *Fumigati*	-	0	-	0	**3.4**	**2**	-	0	Y	Y
*Aspergillus glaucus*	-	0	-	0	**3.3**	**1**	-	0	Rarely	Y
*Aspergillus* section *Nigri*	**26.7**	**1**	-	0	35.0	0	-	0	Y	Y
*Aspergillus* section *Nidulantes*	**25.0**	**1**	-	0	**333.3**	**1**	-	0	Y	Y
*Aspergillus parasiticus*	**275.0**	**2**	-	0	**15.0**	**1**	-	0	Rarely	Y
*Aspergillus* section *Terrei*	**275.0**	**2**	5.0	1	**20.0**	**1**	-	0	Y	Y
*Bisifusarium dimerum species complex*	**107.5**	**1**	-	0	**1.7**	**1**	**1.7**	**1**	Y	Y
*Candida aaseri*	-	0	-	0	**166.7**	**1**	**5.0**	**1**	Rarely	N
*Candida tropicalis*	14.2	1	-	0	**41.7**	**1**	-	0	Y	Rarely
*Cladosporium* sp.	-	0	-	0	**166.7**	**1**	-	0	(very) Rarely	N
*Cladosporium halotolerans*	-	0	-	0	-	0	**1.7**	**1**	N	N
*Colletotrichum* sp.	-	0	-	0	**5.0**	**1**	-	0	Rarely	N
*Emericellopsis fuci*	**1.7**	**1**	-	0	-	0	-	0	(very) Rarely	N
*Exophiala* sp.	**1.7**	**1**	-	0	**17.5**	**2**	-	0	Y	Y^d^
*Fonsecaea pedrosoi*	-	0	-	0	-	0	**1.7**	**1**	Y	Y
*Fusarium* sp.	-	0	**5.0**	**1**	-	0	-	0	Y	Y
*Meyerozyma caribbica*	-	0	-	0	**45.0**	**1**	-	0	Y	N
*Meyerozyma guilliermondii (Candida guillermondii)*	-	0	-	0	**1.7**	**1**	-	0	Y	Y
*Nakaseomyces glabrata (Candida glabrata)*	**16.7**	**1**	-	0	-	0	-	0	Y	Y
*Paecilomyces* sp.	-	0	-	0	-	0	**1.7**	**1**	Y	Y
*Paecilomyces variotti*	-	0	-	0	**16.7**	**1**	-	0	Y	Y
*Penicillum* sp.	**200.0**	**1**	-	0	-	0	**5.0**	**1**	(very) Rarely	N
*Pichia kudriavzevii (Candida krusei)*	**14.2**	**1**	-	0	**100.0**	**1**	-	0	Y	Y
*Pseudallescheria ellipsoidea*	**1.7**	**1**	-	0	-	0	-	0	Y	Y
*Rhodotorula* sp.	-	0	-	0	**20.0**	**1**	-	0	Y	Y
*Talaromyces cnidii*	**166.7**	**1**	-	0	-	0	**1.7**	**1**	N	(Unknown)
*Torula mackenziei*	-	0	-	0	**266.7**	**1**	-	0	Y	Y^e^
*Trichoderma* sp.	-	0	-	0	**1.7**	**1**	-	0	(very) Rarely	Y

^a^Temperature with highest counts of the three (30, 37, 40 °C) was used for reporting for health protection as the worst-case scenario. ^b^Days detected. ^c^“-” = not detected. ^d^Antifungal resistance to echinocandins only. ^e^Antifungal resistance to amphotericin B

**Fig. 4:**
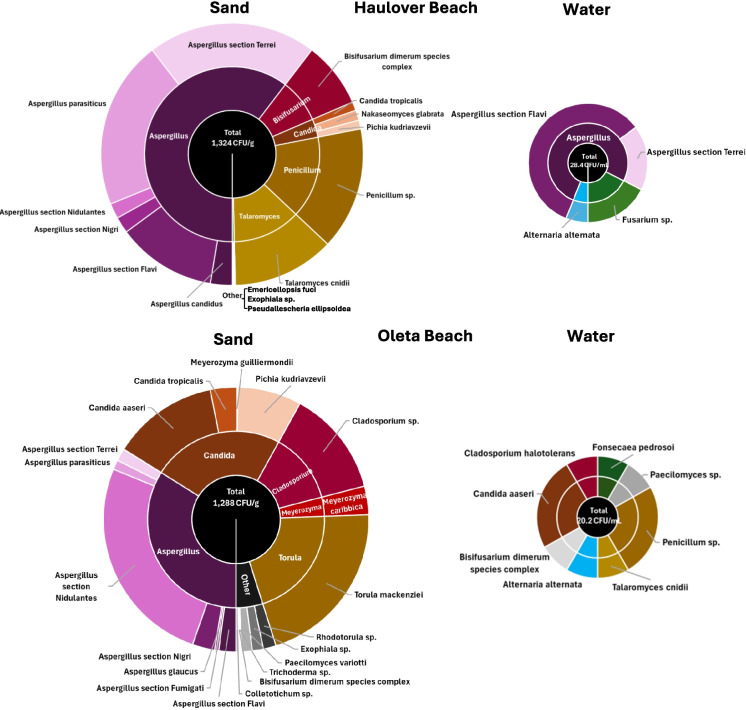
Fungal species found in sand and water at Haulover Beach (top panels), and at Oleta Beach (bottom panels). Sizes of circles are representative of the total number of fungi within each media

When looking at the distribution of species between sand and water, more fungal species were identified in sand compared to water (Fig. 4, compare left to right panels). At Haulover Beach, *Aspergillus* species dominated. Two *Aspergillus* taxa (section *Flavi*., and section *Terrei*.) were observed in both sand and water. Water, however, had only two species that were unique to this matrix (*Alternaria alternata*, which is rather hydrophilic in nature, and *Fusarium* sp.) whereas sand had 13 species that were unique to this matrix, including four unique *Aspergilli* and three unique *Candida* species including *Candida tropicalis*, *Nakaseomyces glabrata* formerly known as *Candida glabrata*, which has a low sensitivity to azoles, and *Pichia kudriavzevii* formerly known as *C. krusei*, intrinsically resistant to azoles*.* Other fungal species observed in high numbers (> 10% of the total) included *Penicillum *sp. and *Talaromyces cnidii.* Fungal species observed in small quantities (< 1% of the total) included *Emericellopsis fuci*, *Exophiala* sp., *Bisifusarium dimerum species complex*, and *Pseudallescheria ellipsoiea*. For Oleta Beach, both *Aspergillus* (*n* = 7 species) and *Candida* species (*n* = 4) dominated in the sand. No *Aspergilli* were observed in the water, and only one *Candida* (*Candida aaseri*) was observed in both the sand and water. *Bisifusarium dimerum species* complex was also observed in both sand and water but at lower levels than *Candida aaseari*. At Oleta, an additional 6 species were unique to water, and 18 were unique species to sand.

**Fig. 5:**
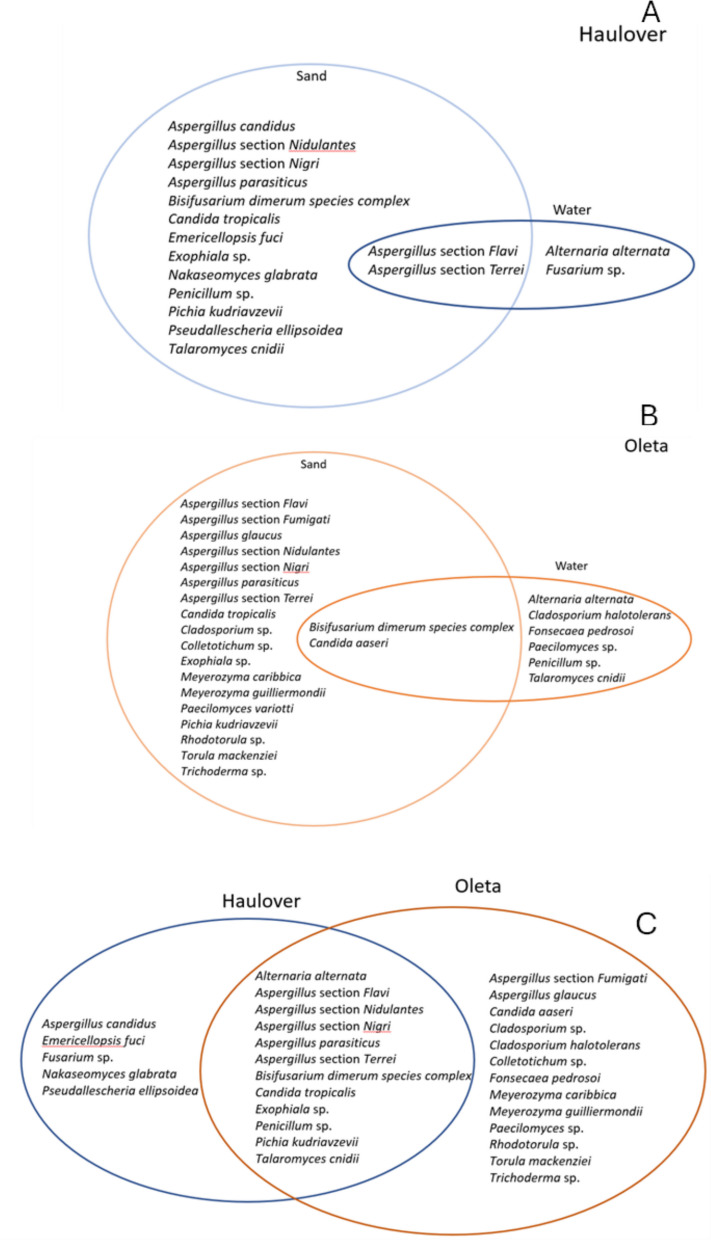
Venn diagram of fungal species found in sand versus water at Haulover Beach (**A**), and at Oleta Beach (**B**) and comparison of fungal species between Haulover and Oleta Beaches (**C**)

Comparisons between Haulover and Oleta showed 12 species (including 5 *Aspergilli* and 2 *Candida*) shared among both beaches (see Table 1 for the complete list of all fungi detected), with Haulover having an additional five unique species and Oleta having an additional 13 unique species (easiest seen in Fig. 5 C). Among the *Candida*, *Nakaseomyces glabrata* (*Candida glabrata*) was unique to the more saline beach of Haulover, whereas *Candida aaseri* and *Meyrozyma guilliermondii* (*C. guillermondii)* were unique to the estuarine beach of Oleta. *Candida* species common among both beaches included *Pichia kudriavzevii* (*C. krusei*) and *C. tropicalis*. Similarly, *Aspergilli* unique to Haulover included *Aspergillus candidus*, and unique to Oleta included *Aspergillus* section *Fumigati* and *Aspergillus glaucus*. *Aspergilli* common to both beaches included *Aspergillus* section *Flavi*, *Aspergillus* section *Nidulantes*, *Aspergillus* section *Nigri*, *Aspergillus parasiticus*, and *Aspergillus* section *Terrei*.

Among the *Aspergillus* section *Fumigati* isolates tested, all were identified as sensu stricto, and no azole resistance was detected. Within the yeasts, *C. auris* was not detected.

## Discussion

### Total fungi levels in sand

The average total fungal counts at both beaches (> 1200 CFU/g) exceeded the average total fungal counts (90 CFU/g) acknowledged by the WHO. These values for the two study beaches were higher than the median total fungi for the marine beaches from the Mycosands I study (coastal beaches, *n* = 660, median = 77 CFU/g, range (0, 3497.5 CFU/g) and freshwater beaches, *n* = 42, median = 201.7, range (0, 6400 CFU/g) (Brandão et al., 2021). The high levels of total fungi at the two study beaches may be due to the warmer, more humid environment of the subtropical study site compared to the sites primarily located in Europe included in the Brandão et al. and’s (2021) study. Higher fungal counts in warmer, more humid environments are expected as fungi prefer moist conditions with an ideal temperature range from 25 to 30 °C for most fungi (Cogliati et al., 2023; Brandão et al. (2022); Dix & Webster, 1995). Also, sands in highly urban areas and impacted by rivers, such as those included in the current study, have been observed to contain elevated levels of total fungi. Examples include freshwater beaches of the Mycosands I initiative and a river-impacted beach in Italy (420 CFU/g, Soffritti et al., 2023).

In contrast to enterococci, the total fungi concentrations in sand and in water were similar for each study beach. Such observations suggest that factors that influence fungi concentrations at beaches are different than those that influence enterococci concentrations (Carducci et al., 2022). Enterococci levels tend to be more strongly influenced by geomorphology, which influences water circulation, with ocean-facing beaches showing lower levels of enterococci relative to bay-facing beaches (Donahue et al., 2017). In the current study, the difference in average total fungal counts between ocean- and bay-facing beaches was minimal. This could be due to impacts to Oleta Beach by the Oleta River and impacts to Haulover Beach from tidal flushing of Biscayne Bay through Baker’s Haulover Cut (Fig. 1). Li et al. (2023), for beaches in China, found that seawater fungal communities shifted with seasons but not with locations, suggesting stronger environmental factors such as temperature contributing to fungal communities. Given the differences between enterococci and fungi measurements, results indicate that enterococci are not capable of capturing all potential microbial risks at recreational beaches. These overall differences in the levels of fungi relative to enterococci underscore the need for public health thresholds that are not based solely on enterococci but also include fungi concentrations.

### Diversity of fungi between sand and water

Higher levels of fungi and greater diversity were observed in sand relative to water. The dominance of total fungal counts in beach sand has been consistently observed at beaches throughout the world, including Europe and Australia (Brandão et al., 2021) and Israel (Frenkel et al., 2022). This is not surprising given the fungal cell wall architecture (Diamond, 1988; Gow & Lenardon, 2023) of most molds, which can serve to bind fungi to sand. Although, on a weight basis, levels in sand were much higher for both enterococci and fungi, the difference in levels between water and sand is the greatest for fungi. Compared to enterococci, fungi are more concentrated in beach sand in comparison to beach water.

In addition to total counts, the higher diversity in fungal species within sand (relative to the water) has been consistently observed in other studies (Brandão et al., 2021; Moazeni et al., 2023). In addition, a relatively small number of fungal species were shared among both sand and water at each beach. Such results suggest limited exchange of fungi at the sediment–water interface. Such exchanges would be expected given the tidal and wave action within the intertidal zone (Feng et al., 2015) and the exchange due to wind-driven sand transport and aerosolization of water through wave action (Abdool-Ghany et al., 2023).

Overall, species identification of fungi at both beaches was dominated by *Aspergillus* and *Candida* spp. consistent with observations for beaches in Europe (Brandão et al., 2021; Gangneux et al., 2024; Novak Babič et al., 2022; Prigitano et al., 2023), Africa (Diop et al., 2024; Migahed, 2003; Selvarajan et al., 2024), and Asia (Lee et al., 2023; Moazeni et al., 2023; Yee et al., 2016). When comparing the fungal species between beaches, a larger number of fungi were shared (12 inclusive of 5 *Aspergillus* species and 2 *Candida* species) (Fig. 5). Fewer species were unique to Haulover (6 species) in comparison to the number that were unique to Oleta (13). The higher number of fungal species observed at Oleta may be related to its location along a brackish river (21.6 psu), which may be influenced more by vegetation and urban sources, in comparison to Haulover, which is marine (34.6 psu) and facing the Atlantic Ocean.

Among the fungi identified in the samples, some are known common pathogens and others rare (Table 1, last two columns). In this study, a total of 25 taxa with reports of causing disease (common and rare) in humans were identified. Among these 25 taxa, 20 have been previously reported to be azole resistant to some or all classes of antifungals available, namely to echinocandins, azoles, and amphotericin B (Prigitano et al., 2021; Kalcanci et al., 2006).

No azole resistance was detected among the *Aspergillus* section *Fumigati* isolates, and three different species of *Candida* were identified (*C. tropicalis*, *C. glabrata*, and *C. krusei*) that have been documented to carry azole resistance. Azole resistance was not measured directly for these isolates, and future work should test all *Candida* species isolated for antifungal resistance.

### Why focus on fungi in the USA

Several pathogenic fungi are already endemic in the USA (Lockhart et al., 2021). However, comprehensive studies are sparse, and more research is needed to document expected levels of fungi in beach environments throughout the USA and the Americas. In the USA, such studies are limited to historical studies in Hawai’i (Dunn & Baker, 1984; Kishimoto & Baker, 1969) and Florida (Fell et al., 1960; Shah et al., 2011) prior to the widespread use of sequencing. In the Caribbean, Central, and South America, studies are specific to a species or smaller group of fungi, such as studies in Brazil (e.g., *C. tropicalis*, Zuza-Alves et al., 2016), in Puerto Rico for filamentous fungi (Echevarría, 2019), and in Mexico for arenicolous marine fungi (communities specific to sand) (Olguin et al., 2023; Velez et al., 2021, 2022).

### Potential approach for integrating fungal measurements into routine monitoring programs

The fungi levels measured in this study may pose a health risk. However, the results of this study do not comprise evidence of this risk. As mentioned in prior sections, potential impacts to human health from pathogenic microbes in the environment are derived by monitoring pathogenic microbes in human exposure zones. Monitoring is the first step of several in the validated traditional health risk assessment, which includes subsequent steps: (2) dose–response, (3) exposure, and (4) microbial risk assessment. Here, we propose an approach for integrating fungi into the first step and make brief recommendations on further research to address information gaps in steps 2 and 4.

Currently, beach water quality is regulated through culture-based measurements of FIB. For consistency with FIB monitoring, culture-based measurements of fungi should also be implemented, possibly through total fungal counts, which may be considered a taxa-blind general indicator of the level of fungal contamination of any matrix. Additionally, incubation at 37 °C can help select pathogenic species with the potential to cause invasive infections. Given their predominance in beaches throughout the world and their link to human health impacts, coupled with results in this study, *Aspergillus* and *Candida* sp. may serve as potential fungal indicators beyond total fungal counts. Technologies should be explored to identify and quantify fungal species, such as PCR methods (Klingspor & Jalal, 2006) and sequencing (Romão et al., 2017) for routine monitoring. One advantage of the Mycosands initiatives is the standardization of methods across laboratories, allowing for more direct comparisons between study sites (Sabino et al., 2014a). When total fungal counts by culture exceed a set threshold, then molecular or mass spectroscopy-based measures can be implemented to identify potentially pathogenic fungal species. Epidemiologic studies and/or quantitative microbial risk assessments (QMRA) (WHO, 2021, Weiskerger & Brandão, 2020) should also be conducted to relate environmental fungal measurements to human health impacts. QMRA should integrate human behavioral factors specific to recreational beach use (Ferguson et al., 2019, 2020, 2021a, b) and dose–response relationships (Gitter et al., 2023; Shibata & Solo-Gabriele, 2012; Hamilton et al., 2024; Benedict et al., 2019). The levels of pathogenic fungi species can then be compared to set thresholds established through epidemiologic studies or QMRA.

## Conclusions

This study evaluated two study beaches (ocean facing and bay facing) as areas for potential exposure to fungal pathogens. Fungal measurements were compared to the FIB enterococci to assess their association and thus determine if FIB measurements alone capture all microbial risks to human health. We used a culture-based method (drawn from the Mycosands II initiative) with higher incubation temperatures to isolate fungi that can cause invasive infection. Enterococci and fungi exceeded guideline levels at the two study beaches, with differences observed between enterococci and fungi for both beaches, suggesting that factors that influence fungi concentrations at beaches are different than those that influence enterococci concentrations. Importantly, compared to enterococci, fungi are more concentrated in beach sand in comparison to beach water. The most common fungal species observed were from the *Aspergillus* and *Candida* genera, with the highest levels and diversity of fungi observed in beach sands, in comparison to water. Among the species identified, eight Aspergillus species (*alternata*, *flavi*, *fumigati*, *glaucus*, *nigri*, *nidulantes*, *parasiticus*, and *terrei*) and four *Candida* species (*tropicalis*, *glabrata*, *guilliermondii*, and *krusei*) are pathogenic with known potential resistance to antifungals. Whereas enterococci exceedances are associated with a risk of gastrointestinal illness, exposure to the most common fungal species identified in this study may cause a range of illnesses from cutaneous and subcutaneous infection, pulmonary infection, and systemic diseases. However, the results of this study do not comprise evidence of this risk. Efforts are needed to standardize the analysis of beach sands for fungi. We recommend using elevated incubation temperatures (37 °C growth in the Sabouraud plates) to improve fungal selectivity and routine testing of *Aspergillus* and *Candida* species for antifungal resistance. Fungal measurements should be related to human health risks through epidemiologic studies and/or quantitative microbial risk assessment such that guideline levels can be enacted and based upon acceptable human health risks. Establishing methods and monitoring programs in the Americas and throughout the world can provide insights into the dynamics of fungal disease transmission from environmental reservoirs.

## Data Availability

Data is provided within the manuscript and supplementary information files. Sequences were deposited in GeneBank database under the following Gene Bank Accession Numbers: PQ863779 - PQ863788.
